# Emerging Technologies in Sports-Related Knee Injuries and Rehabilitation: A Systematic Review

**DOI:** 10.7759/cureus.113286

**Published:** 2026-07-24

**Authors:** Durga A Jagdale, Sandeep Shinde

**Affiliations:** 1 Department of Musculoskeletal Sciences, Krishna College of Physiotherapy, Krishna Vishwa Vidyapeeth, Deemed To Be University, Karad, IND; 2 Department of Musculoskeletal Sciences, Krishna College of Physiotherapy, Krishna Vishwa Vidyapeeth, Deemed to be University, Karad, IND

**Keywords:** anterior cruciate ligament reconstruction, knee injuries, patellar tendinopathy, return to sport, sports physiotherapy

## Abstract

Sports-related knee injuries are among the most common musculoskeletal conditions affecting athletes and frequently result in pain, functional limitations, prolonged rehabilitation, and delayed return to sport. Emerging rehabilitation technologies and advanced physiotherapy interventions have gained increasing attention for improving clinical outcomes following anterior cruciate ligament (ACL) injuries, patellar tendinopathy, and other sports-related knee disorders. This systematic review aimed to evaluate the effectiveness of contemporary rehabilitation strategies in enhancing functional recovery, neuromuscular performance, pain reduction, and return-to-sport outcomes among athletic populations with knee injuries.

A systematic search of the literature was conducted using electronic databases, and randomized controlled trials (RCTs) published between 2021 and 2026 were included according to predefined eligibility criteria. Twelve studies involving athletes with ACL injuries, ACL reconstruction, patellar tendinopathy, and other sports-related knee conditions were selected for qualitative synthesis. The included interventions comprised neuromuscular training with external focus cues, eccentric-oriented strength training, Nordic hamstring exercises, proprioceptive and plyometric training, accelerated rehabilitation protocols, objective criteria-based rehabilitation, progressive tendon-loading exercise therapy, personalized exercise prescription, and injury prevention programs. Methodological quality was assessed using the Cochrane Risk of Bias 2 (RoB 2) tool.

The findings demonstrated that emerging rehabilitation approaches generally produced favorable improvements in knee function, neuromuscular control, balance, strength, pain reduction, postural stability, jumping performance, limb symmetry, and return-to-sport readiness compared with conventional rehabilitation alone. Eccentric loading interventions showed particularly favorable outcomes in athletes with patellar tendinopathy, while neuromuscular and criteria-based rehabilitation programs enhanced functional recovery following ACL reconstruction. Accelerated rehabilitation and individualized exercise prescription approaches also demonstrated promising results for optimizing rehabilitation efficiency and sports participation.

The evidence suggests that integrating emerging rehabilitation technologies and evidence-based physiotherapy interventions into sports injury management can substantially improve clinical and functional outcomes in athletes with knee injuries. Although most included studies demonstrated some concerns regarding risk of bias, the collective findings support the use of innovative rehabilitation strategies to facilitate recovery, enhance athletic performance, and promote safe return to sport. Further high-quality RCTs with longer follow-up periods are required to establish standardized rehabilitation guidelines and determine long-term effectiveness.

## Introduction and background

Sports participation has increased globally due to growing awareness of physical fitness, recreational engagement, and competitive performance. Although sports provide substantial physical and psychological benefits, they are also associated with a high risk of musculoskeletal injuries. Among these, knee injuries are one of the most common and clinically significant conditions affecting athletes because they frequently result in pain, functional limitations, prolonged rehabilitation, delayed return to sport, and increased healthcare costs. The knee joint is exposed to considerable mechanical stress during sprinting, jumping, cutting, pivoting, landing, and rapid deceleration, making athletes particularly susceptible to both acute traumatic injuries and chronic overuse conditions. Consequently, sports-related knee injuries remain a major concern in sports medicine due to their multifactorial nature and impact on athletic performance and long-term joint health [1,2].

Sports-related knee injuries can be broadly categorized into acute traumatic injuries, such as anterior cruciate ligament (ACL), posterior cruciate ligament, collateral ligament, and meniscal tears; and chronic or overuse injuries, including patellofemoral pain syndrome, patellar tendinopathy, cartilage lesions, and osteochondral injuries. ACL injuries are considered one of the most serious and clinically significant sports-related knee injuries. Their long-term consequences include persistent functional impairments, early knee osteoarthritis, and an increased risk of recurrent injury [3].

The occurrence of sports-related knee injuries is multifactorial, resulting from the interaction of intrinsic and extrinsic risk factors. Intrinsic factors include anatomical characteristics, neuromuscular deficits, muscle weakness, proprioceptive impairment, joint laxity, and previous injury, whereas extrinsic factors include playing surface, footwear, training load, and sport-specific demands. Biomechanical studies indicate that many ACL injuries occur during non-contact movements involving sudden deceleration, cutting, pivoting, and poor landing mechanics. Dynamic knee valgus, inadequate knee flexion, altered trunk alignment, and impaired neuromuscular control further increase ACL loading and injury risk [4,5].

**Figure 1:**
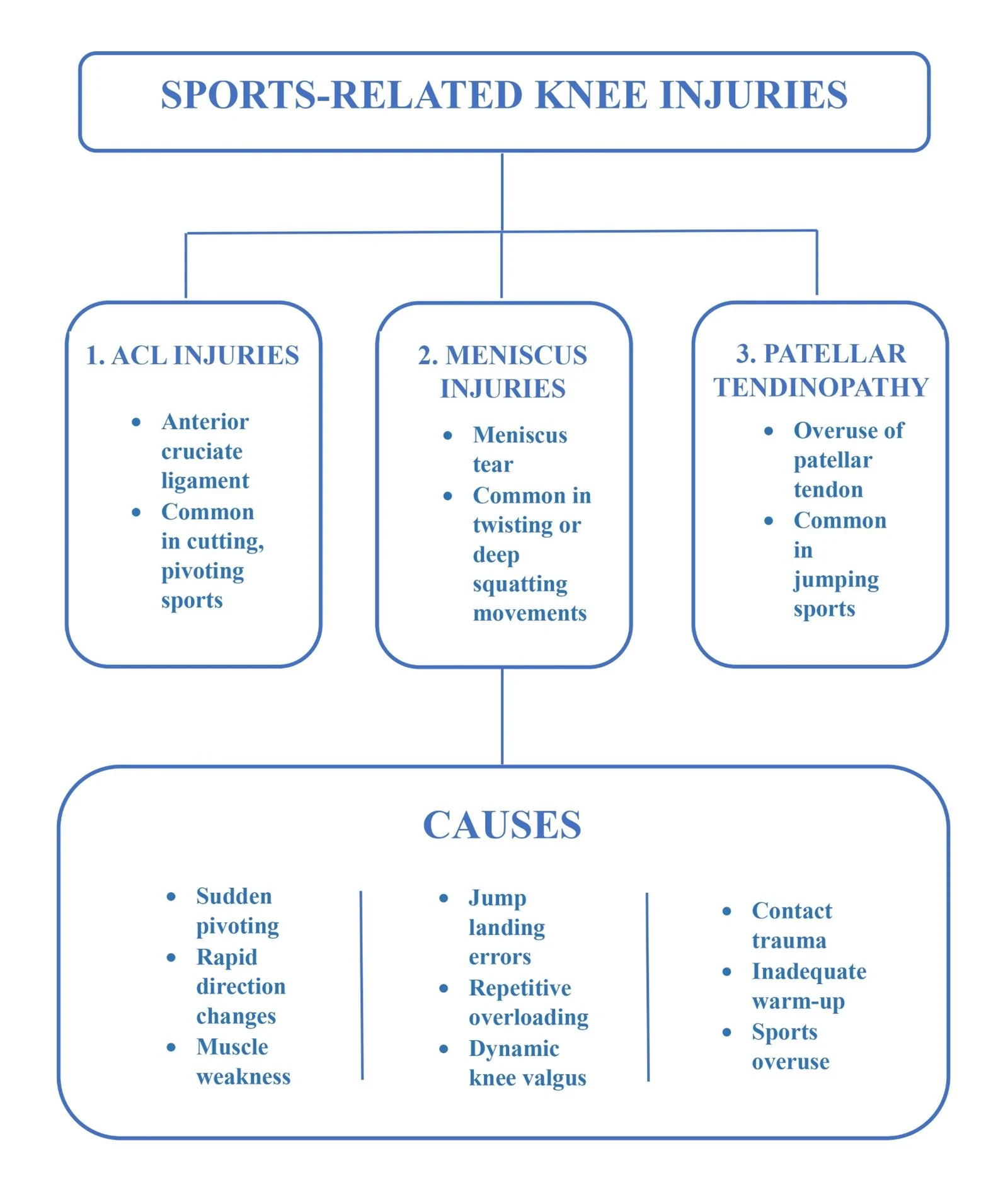
Injuries and causes of sports-related knee injuries ACL: Anterior Cruciate Ligament Image created by Durga A. Jagdale, using Microsoft PowerPoint (Microsoft Corporation, Redmond, WA, USA).

Research on injury mechanisms indicates that many major knee injuries occur in non-contact situations. Ligamentous injuries, especially ACL ruptures, have been found to be frequently caused by sudden deceleration, cutting maneuvers, pivoting motions, and poor landing mechanics. Excessive dynamic knee valgus, decreased knee flexion angles, changed trunk alignment, and insufficient neuromuscular control all enhance joint stress and injury risk, according to biomechanical assessments. The knowledge of modifiable factors linked to sports-related knee injuries has greatly improved as a result of these findings [5,6], as shown in Figure 1.

Specific injury prevention programs have been developed as a result of the identification of modifiable biomechanical and neuromuscular risk factors. Enhancing strength, balance, proprioception, movement quality, agility, and neuromuscular control are the main goals of modern preventative techniques. These therapies are intended to improve dynamic joint stability, rectify abnormal movement patterns, and lessen excessive knee structural loading during sports involvement. Programs for preventive exercise are becoming a crucial part of global efforts to enhance athlete safety and reduce the risk of injury. One of the most effective methods for lowering the risk of knee injuries is neuromuscular training. Plyometric exercises, balance training, strength training, agility drills, and movement retraining methods are frequently included in these programs. Reductions in injury-related risk variables have been linked to improvements in lower-extremity alignment, landing mechanics, postural control, and muscular coordination. As a result, systematic neuromuscular training regimens have been widely accepted in situations related to athletic performance and sports medicine [6,7].

Effective rehabilitation requires an individualized approach that addresses physical, functional, and psychological recovery. Modern rehabilitation extends beyond pain relief and muscle strengthening to include neuromuscular control, movement quality, sport-specific performance, and psychological readiness. Return-to-sport decisions are increasingly guided by objective functional testing, strength assessment, movement analysis, and psychological evaluation rather than time-based criteria alone, thereby reducing the risk of reinjury [7-9].

**Figure 2:**
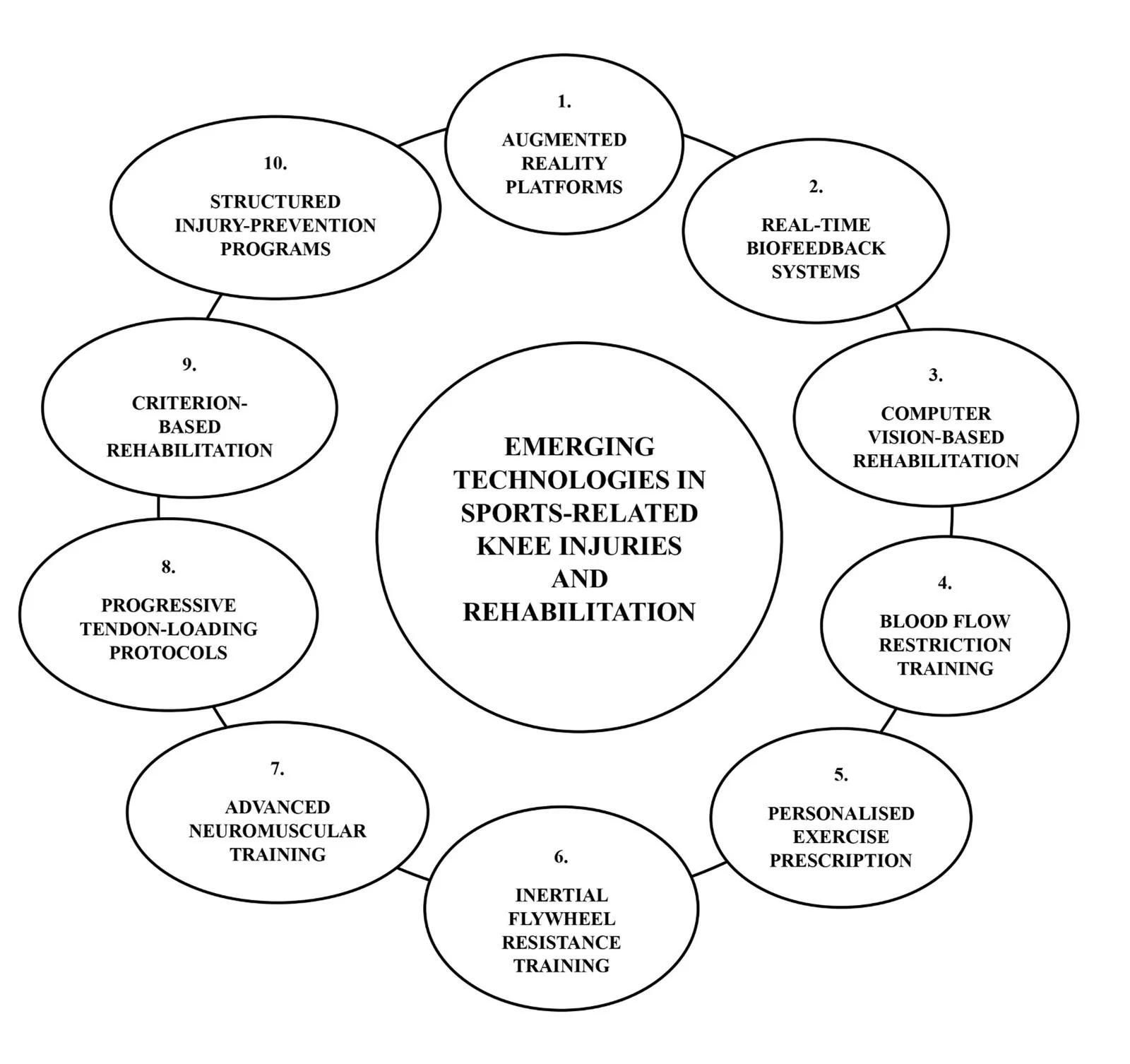
Emerging technologies in sports-related knee injuries Image created by Durga A. Jagdale, using Microsoft PowerPoint (Microsoft Corporation, Redmond, WA, USA).

Recent advances in technology have created new opportunities for improving the assessment, prevention, and rehabilitation of sports-related knee injuries. Emerging technologies are increasingly being incorporated into sports medicine practice and can be broadly categorized into: assessment tools, including wearable sensors, inertial measurement units, motion capture systems, force platforms, and surface electromyography; intervention tools, such as virtual reality, augmented reality, blood flow restriction training, and tele-rehabilitation platforms; and decision-support systems, including artificial intelligence and machine learning algorithms. These innovations provide objective, real-time, and data-driven information that can enhance clinical decision-making and optimize rehabilitation outcomes [10-13], as shown in Figure 2.

Therefore, the primary aim of this systematic review is to evaluate the current evidence regarding exercise-based rehabilitation and emerging technology-assisted interventions for sports-related knee injuries. Specifically, this review focuses on the effects of these interventions on pain, muscle strength, dynamic stability, proprioception, functional performance, athletic readiness, and return-to-sport outcomes. Furthermore, the review aims to investigate the contribution of the entire kinetic chain, including the trunk, hip, knee, and lower extremity musculature, in influencing knee biomechanics and injury recovery. By synthesizing evidence from randomized controlled trials (RCTs), this systematic review seeks to identify the most effective rehabilitation strategies and emerging technologies for improving clinical outcomes, enhancing athletic performance, preventing recurrent injuries, and supporting evidence-based sports rehabilitation practice.

## Review

Methodology

Study Design

To maintain high levels of clarity and scientific integrity, this systematic review followed the Preferred Reporting Items for Systematic Reviews and Meta-Analyses (PRISMA) framework during the data collection and reporting phases [14].

Search Strategy

A systematic literature search was conducted in the Physiotherapy Evidence Database (PEDro), PubMed/Medical Literature Analysis and Retrieval System Online (MEDLINE), Cumulative Index to Nursing and Allied Health Literature (CINAHL), and Scopus to identify relevant articles published from January 2021 to June 2026. The search strategy was developed using a combination of Medical Subject Headings (MeSH) terms (where applicable), free-text keywords, and Boolean operators (AND, OR), with database-specific modifications to optimize retrieval. An example of the PubMed search strategy was: ("Anterior Cruciate Ligament" OR ACL OR "Sports-related knee injuries" OR "Meniscal injury" OR "Patellar tendinopathy") AND ("Rehabilitation" OR Physiotherapy OR "Exercise therapy" OR "Neuromuscular training" OR "Return to sport") AND (Athletes OR Sports). Similar search strategies were adapted for PEDro, CINAHL, and Scopus according to their respective indexing systems. The literature search was restricted to English-language articles published between January 2021 and June 2026.

Eligibility criteria

Inclusion Criteria

Studies were included if they involved athletes or individuals who regularly participate in structured sports, exercise, or recreational physical activities with sports-related knee injuries, including soft tissue injuries such as ACL injuries, meniscal injuries, patellar tendinopathy, or other sports-related knee disorders. Only RCTs were considered. Articles published in English and reporting functional, biomechanical, strength, pain, balance, or return-to-sport outcomes were eligible, as shown in Table 1.

**Table 1:** Eligibility criteria of the included studies ACL: Anterior Cruciate Ligament; RCTs: Randomized Controlled Trials

Criteria	Inclusion Criteria	Exclusion Criteria
Participants	Athletes and physically active individuals	Non-athletic populations only
Joint	Sports-related knee injuries	Non-knee musculoskeletal conditions
Condition	ACL injuries/reconstruction, meniscal injuries, patellar tendinopathy	Hip, ankle, shoulder, or spinal injuries
Intervention	Emerging technology-based rehabilitation interventions	Studies without rehabilitation interventions
Language	English language studies	Non-English studies
Study design	RCTs only	Case reports, case series, reviews, editorials
Outcomes	Pain, stiffness, range of motion, functional outcomes, and muscle strength	Studies without relevant outcome measures
Study population	Human participants	Animal or cadaver studies

Exclusion Criteria

Studies were excluded if they involved non-sports-related knee conditions, knee osteoarthritis without a sports injury component, fractures, postoperative complications unrelated to sports injuries, or other lower-limb injuries not primarily affecting the knee. Reviews, systematic reviews, meta-analyses, case reports, conference abstracts, editorials, letters to the editor, and studies not available in full text were excluded. Studies published in languages other than English were also excluded. Additionally, studies involving animal models or cadaveric research were not considered for this review, as shown in Table 1.

Study Selection

Titles and abstracts were screened against the predefined eligibility criteria, followed by full-text assessment of potentially relevant articles. Any uncertainties regarding study eligibility were resolved through discussion among the reviewers.

Data Extraction

Data extracted from eligible studies included author details, publication year, study design, sample size, interventions, outcome measures, and key findings. Any discrepancies in data extraction were resolved through discussion among the reviewers.

Quality Assessment

Methodological quality and risk of bias were evaluated using study-specific validated tools to address the heterogeneity of the research designs. Specifically, the methodological quality and internal validity of the included RCTs were assessed using the Cochrane Collaboration’s Risk of Bias 2 (RoB 2) tool, which systematically evaluates critical study domains including the randomization process, deviations from intended interventions, missing outcome data, measurement of the outcome, and selection of the reported result [15].

Data Synthesis

Due to substantial heterogeneity in study designs, interventions, outcome measures, follow-up durations, and reporting methods, a narrative synthesis was performed. Quantitative meta-analysis was not considered appropriate because pooling of data could have produced misleading estimates of treatment effects. 

**Figure 3:**
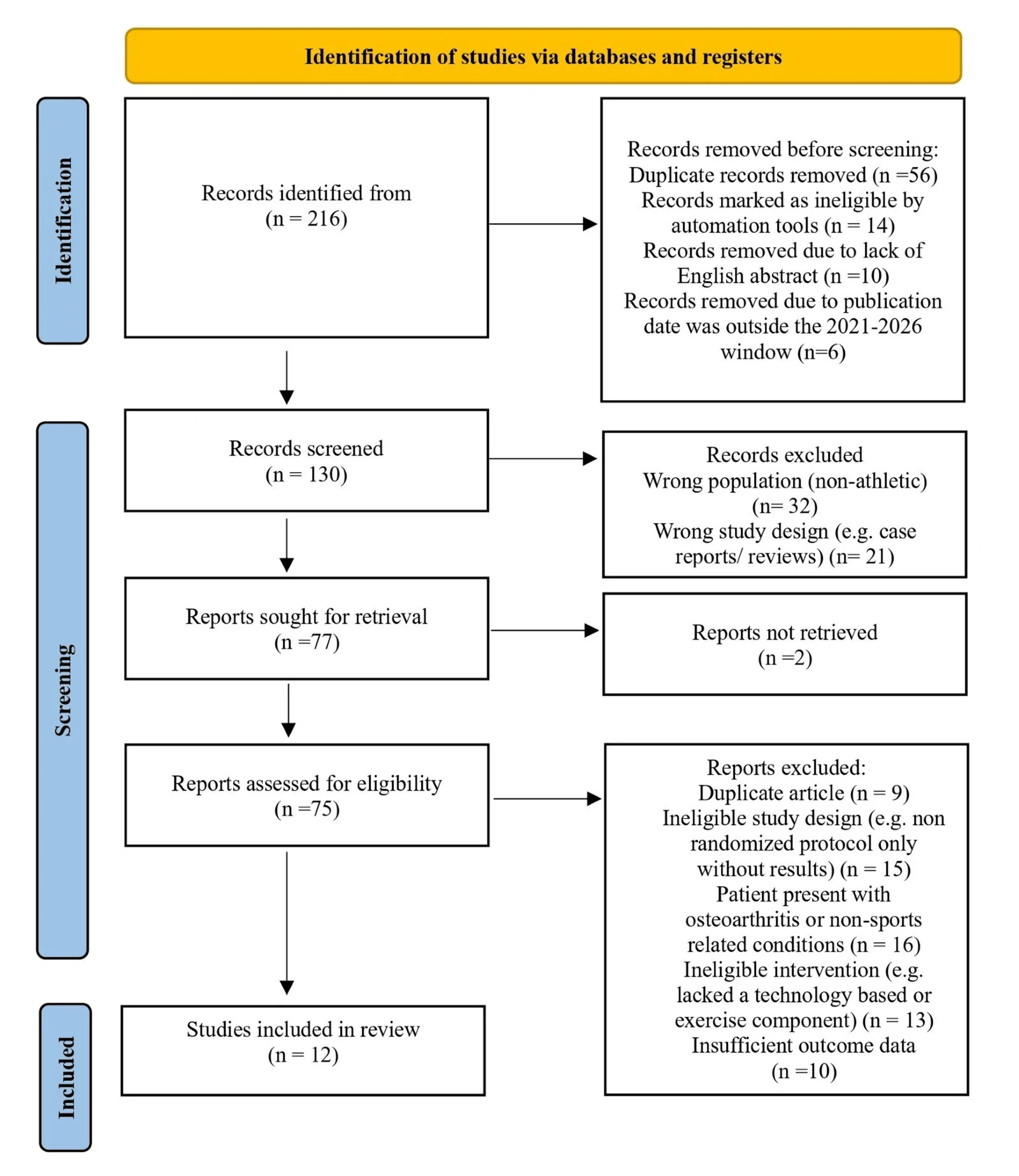
PRISMA flow diagram PRISMA: Preferred Reporting Items for Systematic Reviews and Meta-Analyses

A total of 216 records were identified through database searching. After removing duplicates and other ineligible records, 130 studies were screened. Following title and abstract screening, 75 full-text articles were assessed for eligibility. Sixty-three studies were excluded because of inappropriate duplicate articles, study design, ineligible population or intervention, or insufficient outcome data. Ultimately, 12 studies were included in the systematic review. Figure 3 shows the PRISMA flowchart [14].

The present systematic review included 12 RCTs evaluating rehabilitation and injury prevention strategies for sports-related knee injuries, primarily ACL reconstruction and patellar tendinopathy. Rehabilitation interventions, including neuromuscular training, eccentric-oriented strengthening, progressive tendon-loading exercises, flywheel resistance training, proprioceptive and plyometric training, accelerated rehabilitation, and objective criteria-based rehabilitation, improved pain, muscle strength, balance, neuromuscular control, functional performance, knee stability, and return-to-sport outcomes. Studies on patellar tendinopathy demonstrated improvements in pain, quadriceps strength, postural control, and tendon function, while ACL rehabilitation studies showed enhanced functional recovery and limb symmetry. Injury prevention programmes reduced the prevalence of lower-limb injuries among athletes, and technology-assisted rehabilitation approaches supported objective assessment and individualized rehabilitation progression. Overall, the evidence supports individualized, progressive, and sport-specific rehabilitation programmes to optimize recovery and facilitate a safe return to sport following sports-related knee injuries. Of the included studies, the majority evaluated post-injury rehabilitation interventions, while one study, Lindblom et al.(2023), focused primarily on injury prevention. The details of the 12 included studies are summarized in Table 2.

**Table 2:** Overview of the 12 articles selected for this study ACL : Anterior Cruciate Ligament, ACLR : Anterior Cruciate Ligament Reconstruction, ACL-RSI : Anterior Cruciate Ligament–Return to Sport after Injury Scale, ANT : Anterior Reach Direction, BBS : Berg Balance Scale, BDJ : Bilateral Drop Jump, CG : Control Group, CKC : Closed Kinetic Chain, COHD : Unilateral Triple Crossover Hop for Distance, CoPv : Center of Pressure Velocity, EEG : Eccentric Exercise Group, EET : Eccentric Exercise Therapy, EPI : Exercise Prescription Intervention, FITT-VP : Frequency, Intensity, Time, Type, Volume, and Progression, H Ratio : Hamstring-to-Quadriceps Ratio, HSR : Heavy Slow Resistance Training, IFRT : Inertial Flywheel Resistance Training, IKDC : International Knee Documentation Committee, JPS : Joint Position Sense, KOOS : Knee Injury and Osteoarthritis Outcome Score, LRS : Lysholm Rating Scale, LSI : Limb Symmetry Index, MVIC : Maximum Voluntary Isometric Contraction, NPRS : Numeric Pain Rating Scale, OCBR : Objective Criteria-Based Rehabilitation, OKC : Open Kinetic Chain, OSTRC-O2 : Oslo Sports Trauma Research Center Overuse Injury Questionnaire, PM : Posteromedial Reach Direction, PL : Posterolateral Reach Direction, PTLE : Progressive Tendon-Loading Exercise Therapy, ROM : Range of Motion, RTS : Return to Sport, TCAI : Thigh Circumference Atrophy Index, UCMJ : Unilateral Countermovement Jump, UCR : Usual Care Rehabilitation, UDJ : Unilateral Drop Jump, UTHD : Unilateral Triple Hop for Distance, VAS : Visual Analog Scale, VISA-P : Victorian Institute of Sport Assessment–Patella Questionnaire, vGRF : Vertical Ground Reaction Force, WOMAC : Western Ontario and McMaster Universities Osteoarthritis Index, YBT : Y-Balance Test

Study Number	Title	Authors/ Year	Study Design	Age Range	Sample size	Population/ follow up duration	Comparative Groups	Outcome Measures	Results	Conclusion
1	Effects of a Neuromuscular Training Program Using External Focus Attention Cues in Male Athletes with ACL Reconstruction: A Randomized Clinical Trial	Ghaderi M et al. (2021) [16]	Randomized clinical trial	Approximately 18–35 years (young adult male athletes; mean age ≈ 24–25 years)	24 athletes (Experimental n=12, Control n=12)	Male athletes after primary unilateral ACLR using hamstring tendon autograft. Follow-up duration: eight weeks	1. Experimental Group: Neuromuscular training program with external focus attention cues; 2. Control Group: placebo program/routine sport-specific activities without formal neuromuscular training	1. Biomechanical outcomes assessed during single-leg landing: trunk flexion angle, hip flexion angle, knee flexion angle, knee abduction angle, knee internal rotation angle, knee valgus position, vertical ground reaction force (vGRF), loading rate, anterior tibial shear force, knee extension moment, and knee abduction moment; 2. Proprioception outcomes: Knee JPS error; 3. Functional Outcome: IKDC Subjective Knee Evaluation Form	Improvement in landing biomechanics: The neuromuscular training group demonstrated increased trunk flexion (P = 0.003), hip flexion (P = 0.008), and knee flexion (P = 0.012). 2. Reduction in ACL reinjury risk factors: The intervention group showed significant reductions in knee abduction angle (P = 0.018), knee internal rotation angle (P = 0.022), peak knee extension moment (P = 0.022), peak knee abduction moment (P = 0.014), and anterior tibial shear force (P = 0.018). 3. Ground reaction forces: Peak vertical ground reaction force was reduced (P = 0.008). 4. Improvement in knee proprioception: Significant improvement in knee proprioception was observed (P = 0.001), indicating enhanced neuromuscular control of the reconstructed knee.	This randomized clinical trial demonstrated that an eight-week neuromuscular training program using external focus attention cues significantly improved landing biomechanics, knee proprioception, and self-reported knee function in male athletes following ACLR. The intervention increased trunk, hip, and knee flexion during landing while reducing knee valgus, internal rotation, anterior tibial shear force, knee moments, and vertical ground reaction forces. These adaptations are associated with a lower risk of secondary ACL injury. Additionally, clinically meaningful improvements in IKDC scores and proprioception were observed. The findings support incorporating externally focused neuromuscular training into post-ACLR rehabilitation and return-to-sport programs.
2	Effectiveness of progressive tendon-loading exercise therapy in patients with patellar tendinopathy: a randomised clinical trial	Breda SJ et al. (2021) [17]	RCT	18–35 years; mean age: 24 ± 4 years	76 participants (58 participants (76%) were male)	Recreational, competitive, and professional athletes with patellar tendinopathy. Follow-up duration: 24 weeks	PTLE group: 38 participants; EET group: 38 participants	1. Primary outcome: VISA-P score; 2. Secondary outcomes: VAS pain during exercise	1. PTLE demonstrated significantly greater improvement compared with EET after 24 weeks (p = 0.023). 2. Pain during exercise was significantly lower in the PTLE group at 24 weeks (p = 0.006). 3. PTLEs produced superior clinical outcomes, greater functional improvement, and lower exercise-related pain compared with EET in patients with patellar tendinopathy.	The study concluded that PTLE therapy is superior to EET for the conservative treatment of patellar tendinopathy. PTLE resulted in significantly better VISA-P outcomes and lower pain during rehabilitation exercises. The authors recommended PTLE as the preferred first-line conservative treatment for patellar tendinopathy.
3	Inertial flywheel vs heavy slow resistance training among athletes with patellar tendinopathy: a randomised trial	Ruffino D et al. (2021) [18]	RCT	18–35 years; mean age approximately mid-20s	48 athletes	Competitive and recreational athletes diagnosed with patellar tendinopathy. Follow-up duration: 12 weeks	1. Flywheel Training Group: IFRT; 2. HSR Group: HSR training	Primary outcome: 1. VISA-P score; Secondary Outcomes: 1. VAS pain, 2. Quadriceps strength, 3. Countermovement jump performance, 4. Tendon stiffness and biomechanics, 5. Return-to-sport participation	IFRT was at least as effective as heavy slow resistance training for improving pain and function in athletes with patellar tendinopathy and may provide additional benefits for explosive athletic performance. Significant improvements over time (P < 0.05); no significant between-group differences for primary outcomes (P > 0.05)	The study concluded that both IFRT and HSR training are effective rehabilitation strategies for patellar tendinopathy. However, inertial flywheel training may provide additional improvements in eccentric strength and explosive athletic performance, making it particularly useful for jumping and power-based athletes.
4	Effects of Eccentric-Oriented Strength Training on Return to Sport Criteria in Late-Stage Anterior Cruciate Ligament (ACL)-Reconstructed Professional Team Sport Players	Stojanovic MDM et al. (2023) [19]	RCT	18–35 years	Total participants: ~50 athletes	Professional/elite team sport athletes post ACLR. Follow-up duration: six weeks	1. Experimental group: eccentric-oriented strength training; high-load eccentric exercises for the quadriceps and hamstrings; 2. Control group: standard progressive resistance training/conventional rehab strengthening	1. Single-leg hop for distance; 2. Triple hop; 3. Crossover hop; 4. 6-meter timed hop	1. The eccentric training group showed a greater improvement in quadriceps strength symmetry. Better hop test performance; greater proportion of athletes meeting RTS criteria; 2. Control group: Slower improvement in functional symmetry. Eccentric training improved functional readiness for return to sport more effectively. P < 0.05 for most return-to-sport outcomes.	Eccentric-oriented strength training in late-stage ACL rehabilitation: Improves strength symmetry, enhances functional hop performance, and increases likelihood of meeting RTS criteria. It is recommended as an effective progression strategy in elite and professional ACL rehabilitation programs, particularly in the final phase before return to competition.
5	Extended Knee Control programme lowers weekly hamstring, knee and ankle injury prevalence compared with an adductor strength programme or self-selected injury prevention exercises in adolescent and adult amateur football players: a two-armed cluster-randomised trial with an additional comparison arm	Lindblom H et al. (2023) [20]	RCT	14–46 years	A total of 502 players from 46 teams were included: Extended Knee Control group (n = 197), Adductor programme group (n = 125), and Comparison group (n = 180).	Amateur adolescent and adult male and female football (soccer) players. Follow-up duration: seven months	Experimental group: Extended Knee Control programme, consisting of a running warm-up and six neuromuscular and strengthening exercises with 10 progression levels each, targeting lower-extremity injury prevention. 2. Comparison group 1: Adductor Strength Programme, focused primarily on groin injury prevention. 3. Comparison group 2: Teams already performing self-selected injury prevention exercises (usual practice).	Weekly online injury surveillance questionnaires based on the OSTRC-O2. 2. Exposure hours: Training and match play. 3. Injury incidence: Number of injuries per 1,000 football hours. 4. Injury prevalence: Prevalence rates during the season.	The Extended Knee Control programme appears to provide broader lower-limb protection because it combines: 1. Strength training, 2. Neuromuscular control, 3. Balance exercises, 4. Progressive loading, and 5. Functional movement training. These components likely improved lower-extremity biomechanics and reduced injury risk across multiple body regions, unlike the Adductor Strength Programme, which primarily targeted the groin muscles. A significant reduction in the weekly prevalence of hamstring, knee, and ankle injuries was observed (P < 0.05).	The Extended Knee Control programme was more effective than self-selected injury prevention exercises in reducing hamstring, knee, and ankle injuries among adolescent and adult amateur football players. Although injury incidence did not differ significantly from the Adductor Strength Programme, the Extended Knee Control programme resulted in significantly lower weekly injury prevalence, fewer time-loss injuries, and fewer substantial injuries. The Adductor Programme did not reduce groin injury incidence. Therefore, Extended Knee Control is recommended as a comprehensive injury-prevention strategy for amateur football players
6	Nordic hamstring exercises in functional knee rehabilitation after anterior cruciate ligament reconstruction: a prospective, randomised, controlled study	Chen J et al. (2023) [21]	RCT	18–35 years	60 participants	Patients undergoing rehabilitation after ACLR, Young adult athletic population. Follow-up duration: 12 weeks	1. Experimental Group: Standard ACLR+ Nordic hamstring exercise programme, 2.Control Group: Standard ACLR only.	1. Isokinetic hamstring strength, 2. H:Q ratio, 3. Knee function scores (Lysholm/IKDC), 4. Single-leg hop performance tests, 5. Dynamic balance assessment, 6. Pain assessment using VAS	Eccentric hamstring strength: The Nordic hamstring exercise group demonstrated significantly greater improvements in eccentric hamstring strength compared with the control group (P < 0.05). 2. Functional performance: Participants in the intervention group showed superior improvement in single-leg hop performance, knee stability, and functional knee scores. 3. Hamstring-to-quadriceps strength ratio: A significant improvement in the hamstring-to-quadriceps strength ratio was observed in the Nordic hamstring exercise group, indicating improved muscular balance around the knee joint. 4. Dynamic balance and neuromuscular control: Dynamic balance and neuromuscular control improved more in the intervention group than with standard rehabilitation alone. Overall, significantly improved knee function, hamstring strength, and functional performance were observed after ACLR (P < 0.05).	The study concluded that incorporating Nordic hamstring exercises into ACL rehabilitation programmes significantly improves hamstring strength, neuromuscular control, knee stability, and functional recovery after ACLR. The authors recommended Nordic hamstring exercises as an effective adjunct to standard ACL rehabilitation, particularly for athletes preparing for return to sport.
7	The Effect of Contralateral Knee Neuromuscular Exercises on Static and Dynamic Balance, Knee Function, and Pain in Athletes Who Underwent Anterior Cruciate Ligament Reconstruction: A Single-Blind Randomized Controlled Trial	Karimijashni M et al. (2023) [22]	RCT	Mean age approximately 22–28 years (young athletic population)	40 athletes	Athletes who underwent unilateral ACLR. Follow-up duration: eight weeks)	1. Experimental Group: Conventional ACL rehabilitation + contralateral knee neuromuscular exercises, 2. Control Group: Conventional ACL rehabilitation only	Static balance tests. 2. Dynamic balance tests (Y-Balance Test). 3. KOOS or functional knee scores. 4. VAS for pain.	Balance: The experimental group demonstrated significantly greater improvements in both static and dynamic balance compared with the control group (P < 0.05). 2. Functional knee scores: Functional knee scores improved significantly in both groups; however, the improvement was greater in the contralateral neuromuscular exercise group. 3. Pain: Pain intensity decreased in both groups; however, the experimental group demonstrated superior pain reduction following the intervention. 4. Overall outcomes: Adding contralateral neuromuscular exercises to standard ACL rehabilitation improved postural control, dynamic balance performance, knee function, and pain outcomes more effectively than conventional rehabilitation alone.	The study concluded that incorporating contralateral knee neuromuscular exercises into ACL rehabilitation programmes significantly improves balance, knee function, and pain outcomes in athletes after ACLR. The authors recommended integrating bilateral neuromuscular rehabilitation strategies to optimize recovery and functional performance following ACL surgery.
8	The applied study to improve the treatment of knee sports injuries in ultimate frisbee players based on personalized exercise prescription: a randomized controlled trial	Chen S et al. (2024) [23]	RCT	18–45 years	Initially 118 participants randomized; final analyzed sample = 76 participants (38 intervention, 38 control)	Ultimate frisbee players with knee meniscus injuries. Follow-up duration: six months)	1. Control Group: Standard conservative treatment including drug therapy, physical therapy, rest, and educational materials. 2. EPI Group: Standard treatment plus individualized exercise prescription based on FITT-VP principles.	Primary outcomes: 1. ROM. 2. TCAI. 3. LRS. 4. VAS for pain. Secondary outcome: 1. IKDC score.	Long-term outcomes: Significant long-term improvements remained in the intervention group (P < 0.05). 2. Lysholm and VAS scores: Differences in Lysholm and VAS scores became statistically non-significant by six months, although both groups improved overall. 3. Overall functional outcomes: Personalized exercise prescription significantly improved knee joint mobility, muscle preservation, pain reduction, and knee functional scores compared with standard treatment alone. 4. Rehabilitation outcomes: The intervention group demonstrated superior recovery of knee function and better rehabilitation outcomes throughout follow-up.	The study concluded that personalized exercise prescription is an effective rehabilitation strategy for knee sports injuries in ultimate frisbee players. The intervention reduced pain, improved joint function, minimized thigh muscle atrophy, and enhanced overall athletic recovery compared with conventional treatment alone. The authors recommended individualized exercise-based rehabilitation as an important component of sports injury management.
9	A Comparison between Open Kinetic and Closed Kinetic Chain Exercises Along with Conservative Treatment in Grade-I ACL Injury in Sprinters: A Randomized Controlled Trial	Kumar M et al. (2024) [24]	RCT	18–25 years	50 Male sprinters	Male sprinters diagnosed with Grade-I ACL injury. Follow-up duration: six weeks)	Group A: OKC exercise group. 2. Group B: CKC exercise group.	ROM of the knee joint. 2. NPRS. 3. KOOS.	1. Both groups showed improvement in pain, ROM, and functional outcomes after rehabilitation (p < 0.05). 2. The CKC group demonstrated superior improvement compared to the OKC group in knee ROM and functional recovery in sprinters with Grade-I ACL injury. 3. The mean NPRS score improved in both groups, indicating a reduction in pain intensity. 4. Mean knee ROM after treatment was greater in the CKC group (118 ± 5°) compared with the OKC group (107.2 ± 4.52°). 5. KOOS scores improved significantly in both groups, with better functional gains observed in the CKC group.	The study concluded that both OKC and CKC exercises are beneficial in the rehabilitation of Grade-I ACL injury in sprinters; however, CKC exercises were more effective in improving knee ROM, reducing pain, and enhancing functional outcomes. Therefore, CKC exercises should be preferentially incorporated into rehabilitation protocols for Grade-I ACL injuries in athletes and sprinters.
10	Functional Outcomes of Accelerated Rehabilitation Protocol for ACL Reconstruction in Amateur Athletes: A Randomized Clinical Trial	Elabd OM et al. (2024) [25]	Randomized controlled clinical trial	18–35 years (Mean age: 22.01 ± 1.79 years)	100 participants randomly allocated into two groups (50 Experimental, 50 Control).	Amateur male athletes following ACLR. Follow-up duration: 22 weeks)	1. Experimental Group- Accelerated Rehabilitation Protocol + Conventional Rehabilitation Program. 2. Control Group- Conventional Rehabilitation Program Alone	1. Visual Analogue Scale (VAS) 2. Knee Injury and Osteoarthritis Outcome Score (KOOS) 3. Hop Test Battery- Vertical Jump, Hop for Distance, Drop Jump + Double Hop, Square Hop, Side Hop	1. Pain Reduction (VAS): Greater reduction in pain was observed in the accelerated rehabilitation group. 2. KOOS Functional Outcomes -Improved in both groups. No significant between-group difference. 3. Hop Performance- The Accelerated Rehabilitation Group demonstrated significantly greater improvements in: Vertical jump performance, Hop for distance, Drop jump performance, Square hop performance, Side hop performance. (p < 0.05).	The Accelerated Rehabilitation Protocol was significantly more effective than conventional rehabilitation for improving functional outcomes following ACLR in amateur athletes. Participants receiving the accelerated program achieved better functional performance, greater limb symmetry, improved sports participation capacity, enhanced quality of life, and reduced knee symptoms over the 22-week rehabilitation period. The findings support the use of criterion-based accelerated rehabilitation to optimize recovery and facilitate return to sport after ACLR.
11	Effects of 2 different rehabilitation programs on jumping performance after ACL reconstruction: A randomized controlled trial	Jauregui Bidegain et al. (2025) [26]	RCT	Mean age: 24 ± 6.9 years	40 participants (30 males, 10 females)	Recreational athletes following primary ACLR. Follow-up duration: six and 12 months)	OCBR group. 2. UCR group.	Vertical and horizontal jumping performance. 2. BDJ. 3. UDJ. 4. UCMJ. 5. UTHD. 6. COHD.	Recovery outcomes: OCBR produced superior recovery of explosive lower-limb function after ACLR (P < 0.05). 2. Rehabilitation progression: Functional progression based on biomechanical and strength milestones was more effective than traditional time-based rehabilitation.	The study concluded that: 1. OCBR resulted in significantly better vertical and horizontal jumping performance than UCR after ACLR. 2. Improvements were especially evident at six and 12 months postoperatively. 3. OCBR also enhanced limb symmetry and allowed more patients to safely achieve higher functional levels earlier in rehabilitation. 4. The findings support implementing individualized, criteria-driven rehabilitation protocols to optimize recovery and athletic performance after ACLR.
12	Progressive tendon-loading eccentric exercise therapy in athletes with patellar tendinopathy improves postural control, quadriceps strength, and pain: A randomized clinical trial	Fendri T et al. (2026) [27]	RCT	Not specifically mentioned	40 athletes initially randomized; 30 completed final analysis after withdrawals	Athletes with patellar tendinopathy from volleyball, basketball, and handball teams. Follow-up duration: 12 weeks	1. Group A: EEG, 2. Group B: Control Group, continued regular sports activities without eccentric rehabilitation	Static postural control: CoP v using stabilometric platform. 2. Dynamic postural control: YBT. 3. Quadriceps isometric strength: Dynamometer (MVIC). 4. Pain: VAS and VISA-P questionnaire.	Postural control: Significant reduction in CoPv after intervention in EEG only, indicating better postural control. 2. Dynamic balance: Significant improvement in YBT reach distances and composite score. 3. Quadriceps strength: Significant improvement in quadriceps strength (mean difference = 99 N). 4. Pain: Significant reduction in pain (mean difference = 20.24 points). 5. Balance conditions: Improvements were mainly observed under eyes-closed balance conditions (P < 0.05).	Progressive eccentric decline squat rehabilitation effectively improved postural control, quadriceps strength, and pain outcomes in athletes with patellar tendinopathy. The study supports eccentric tendon-loading exercises as an effective rehabilitation strategy for patellar tendinopathy.

Risk of Bias Assessment

To ensure the credibility of the synthesized data, a risk of bias evaluation was performed for each selected RCT. The methodological quality and internal validity of the selected literature were scrutinized using the RoB2 tool. This validated instrument assesses five core methodological domains: the randomization process, deviations from intended interventions, missing outcome data, measurement of the outcome, and selection of the reported result [15].

The methodological quality of the 12 included RCTs was assessed using the RoB 2 tool. Ten were rated as having some concerns, while 2 were classified as high risk of bias. None of the included studies achieved a consistently “Low Risk” rating across all five RoB 2 domains due to inherent limitations in exercise-based rehabilitation trials, particularly the inability to blind participants and therapists, and the frequent use of subjective or self-reported functional outcome measures. Most studies were rated as having “Some Concerns”, primarily due to performance bias arising from the nature of physiotherapy interventions, limited blinding of outcome assessors in some trials, and occasional lack of detailed reporting of allocation concealment and pre-registered protocols. Two studies were classified as having “High Risk” of bias, mainly due to unclear randomization procedures, incomplete outcome data, and inadequate blinding of outcome assessment, which may have influenced the reliability of reported functional and clinical outcomes, as given in Table 3.

**Table 3:** Risk of bias assessment of randomized controlled trials using Cochrane Collaboration’s Risk of Bias 2 (RoB 2) tool

Study	Bias Arising From the Randomization Process	Bias Due to Deviations From Intended Interventions	Bias Due to Missing Outcome Data	Bias in Measurement of the Outcome	Bias in Selection of the Reported Result	Overall Risk of Bias
Ghaderi M et al. [16]	Low risk	Low risk	Low risk	Some concerns	Some concerns	Some concerns
Breda SJ et al. [17]	Low risk	Low risk	Low risk	Some concerns	Some concerns	Some concerns
Ruffino D et al. [18]	Low risk	Some concerns	Low risk	Some concerns	Some concerns	Some concerns
Stojanovic MDM et al. [19]	Low risk	Low risk	Low risk	Some concerns	Some concerns	Some concerns
Lindblom H et al. [20]	Low risk	Some concerns	Low risk	Some concerns	Some concerns	Some concerns
Chen J et al. [21]	Low risk	Low risk	Low risk	Some concerns	Some concerns	Some concerns
Karimijashni M et al. [22]	Low risk	Low risk	Low risk	Some concerns	Some concerns	Some concerns
Chen S et al. [23]	Some concerns	Some concerns	Some concerns	High risk	Some concerns	High risk
Kumar M et al. [24]	Some concerns	Some concerns	Some concerns	High risk	Some concerns	High risk
Elabd OM et al. [25]	Low risk	Low risk	Low risk	Some concerns	Some concerns	Some concerns
Jauregui Bidegain L et al. [26]	Low risk	Low risk	Low risk	Some concerns	Some concerns	Some concerns
Fendri T et al. [27]	Low risk	Low risk	Low risk	Some concerns	Some concerns	Some concerns

Discussion

Sports-related knee injuries continue to be a significant problem for athletic populations because they frequently lead to long-term functional losses, recurrent injury risk, and extended absences from activity. The results of 12 RCTs examining novel rehabilitation techniques for patellar tendinopathy and ACL injuries in sports populations were compiled in this systematic review. When compared to traditional rehabilitation methods, the evidence showed that modern rehabilitation techniques that prioritize neuromuscular retraining, eccentric strengthening, progressive tendon loading, objective criteria-based progression, and customized exercise prescription result in better improvements in pain, strength, balance, functional performance, and return-to-sport outcomes [17-27]. The results demonstrate how sports physiotherapy has evolved from symptom-focused treatment to performance-oriented rehabilitation with the goal of regaining athletic function and lowering the risk of reinjury.

ACL Rehabilitation

Neuromuscular training following ACL reconstruction consistently improved knee function, movement quality, and athletic performance. Ghaderi et al. reported that incorporating an external focus of attention into neuromuscular training enhanced movement control and functional outcomes, supporting motor learning principles that emphasize automatic movement patterns and improved dynamic knee stability after ACL reconstruction [16].

Progressive strengthening interventions also demonstrated favorable outcomes. Stojanović et al. found that eccentric-oriented strength training improved muscle strength, limb symmetry, and return-to-sport performance, reinforcing the role of progressive resistance training during late-stage rehabilitation [19]. Similarly, Chen et al. showed that Nordic hamstring exercises enhanced knee stability, hamstring strength, and functional performance, highlighting the contribution of hamstring function to reducing anterior tibial translation and supporting ACL stability [21]. Novel rehabilitation strategies also demonstrated promising results. Karimijashni et al. reported that contralateral neuromuscular training improved knee function, pain, and balance through cross-education, offering a useful strategy when loading of the injured limb is limited during early rehabilitation [22].

Criterion-based rehabilitation approaches were consistently associated with superior functional recovery. Elabd et al. demonstrated that accelerated rehabilitation improved dynamic stability, hop performance, limb symmetry, and return-to-sport readiness compared with conventional rehabilitation [25]. Likewise, Jauregui Bidegain et al. reported sustained improvements in jumping performance, neuromuscular control, and return-to-sport readiness, supporting progression based on objective functional milestones rather than time alone [26].

Exercise selection also influenced rehabilitation outcomes. Kumar and Madaan found that both open and closed kinetic chain exercises improved pain and function, although closed kinetic chain exercises produced greater gains in knee function and range of motion. Improved hip and trunk control during these exercises may reduce dynamic knee valgus and ACL loading, supporting their inclusion in early-stage ACL rehabilitation. Clinically, incorporating exercises that target hip abductors, external rotators, and trunk stability alongside closed kinetic chain training may help optimize movement mechanics and reduce the risk of reinjury while facilitating a safe return to sport [24].

Patellar Tendinopathy Rehabilitation

Patellar tendinopathy was the second major condition evaluated in this review. Breda et al. demonstrated that progressive tendon-loading exercise significantly improved pain and functional outcomes, supporting its role in enhancing tendon remodeling and load tolerance [17]. Similarly, Ruffino et al. reported clinically meaningful improvements with both inertial flywheel resistance and heavy slow resistance training, suggesting that different progressive loading strategies can effectively restore tendon function while meeting sport-specific demands [18]. Furthermore, Fendri et al. found that progressive eccentric exercise improved quadriceps strength, pain, and postural control, reinforcing the importance of eccentric loading in optimizing both physical and functional recovery in athletes with patellar tendinopathy [27].

Injury Prevention

One included cluster-randomized trial by Lindblom et al. focused primarily on ACL injury prevention rather than post-injury rehabilitation and was considered separately. When compared to other preventive measures, the Extended Knee Control program dramatically decreased the frequency of knee, hamstring, and ankle injuries among amateur football players. These results highlight the effectiveness of structured neuromuscular injury-prevention programs in lowering the likelihood of lower-extremity injuries in athletes. Therefore, prevention programs that include strength, balance, coordination, and movement-quality activities may be crucial in lowering the burden of future rehabilitation and increasing athlete availability during competitive seasons [20].

Individualized and Technology-Assisted Rehabilitation

The concept of individualized rehabilitation was explored by Chen et al., who investigated personalized exercise prescription among ultimate frisbee players with sports-related knee injuries. By customizing rehabilitation exercises based on individual deficiencies, sports demands, and recovery objectives, personalized therapies achieved positive clinical outcomes. The new paradigm of precision rehabilitation, which acknowledges that athletes may react differently to standardized regimens, is supported by these findings. Therefore, tailored exercise recommendations may improve the efficacy of rehabilitation and maximize functional outcomes in a variety of athletic populations [23].

Collectively, the evidence synthesized in this review demonstrates that emerging rehabilitation technologies and advanced exercise-based interventions can substantially improve outcomes in athletes with sports-related knee injuries. Across ACL reconstruction and patellar tendinopathy populations, interventions emphasizing eccentric strengthening, neuromuscular retraining, objective progression criteria, proprioceptive training, and individualized exercise prescription consistently produced favorable results [17-27]. These approaches address the multifactorial impairments associated with sports knee injuries and facilitate a more comprehensive recovery process than traditional rehabilitation alone.

Clinical Implications

Clinically, the findings of this systematic review suggest that incorporating emerging rehabilitation strategies such as neuromuscular training, eccentric-oriented strengthening, Nordic hamstring exercises, proprioceptive and plyometric training, accelerated rehabilitation protocols, and objective criteria-based rehabilitation can enhance functional recovery following sports-related knee injuries, particularly ACL reconstruction and patellar tendinopathy. For sports medicine providers, integrating progressive strength, neuromuscular, balance, and sport-specific exercises into ACL rehabilitation can improve knee function, stability, and return-to-sport readiness while helping reduce the risk of reinjury. Clinicians should consider integrating individualized, sport-specific, and criteria-driven rehabilitation approaches alongside conventional physiotherapy to optimize recovery outcomes, reduce reinjury risk, and facilitate a safe return to athletic participation.

Limitations

Current research tends to concentrate on short-term rehabilitation outcomes, while there is a relative lack of studies focusing on the long-term effects of exercise therapy, improvements in quality of life, and prevention of re-injury. The included studies demonstrated considerable heterogeneity in interventions and outcome measures; characteristics also prevent the establishment of a definitive gold standard for exercise combinations. Similarly, the eligibility criteria exclusively favoured RCTs; while this ensured a high level of methodological rigor, it excluded lower-level evidence that may provide clinically relevant insights. Additional limitations include the small sample sizes, absence of a meta-analysis due to study heterogeneity, the restriction to English-language publications, and the inclusion of studies with some concerns or a high risk of bias, which may affect the generalizability and certainty of the findings.

Future Directions

Future research should prioritize large-scale, high-quality RCTs with standardized rehabilitation protocols, longer follow-up periods, and consistent outcome measures to strengthen the evidence base. Additionally, studies should evaluate the long-term effectiveness of emerging rehabilitation technologies, such as wearable sensors, artificial intelligence, and tele-rehabilitation, while reporting comprehensive statistical outcomes to facilitate future meta-analyses and evidence-based clinical recommendations.

## Conclusions

Sports-related knee injuries continue to represent a significant challenge in athletic populations due to their high incidence, prolonged recovery periods, and substantial risk of reinjury. The evidence indicates that contemporary rehabilitation approaches incorporating emerging technologies and innovative exercise interventions can significantly enhance recovery outcomes. Advanced strategies such as neuromuscular training, real-time biofeedback, blood flow restriction training, progressive tendon-loading protocols, inertial flywheel resistance training, personalized exercise prescription, and structured injury-prevention programs demonstrated positive effects on pain reduction, muscle strength, proprioception, balance, functional performance, and return-to-sport readiness. These interventions promote a more individualized, objective, and function-oriented approach to rehabilitation, thereby optimizing recovery and athletic performance. Furthermore, technology-assisted rehabilitation may improve movement quality, enhance neuromuscular control, and contribute to reducing the risk of recurrent injury. While the current evidence supports the integration of these innovative approaches into sports rehabilitation practice, further high-quality RCTs with standardized outcome measures and long-term follow-up are warranted to establish their long-term effectiveness and clinical applicability.
